# Feature Transformation for Efficient Blood Glucose Prediction in Type 1 Diabetes Mellitus Patients

**DOI:** 10.3390/diagnostics13030340

**Published:** 2023-01-17

**Authors:** Hatim Butt, Ikramullah Khosa, Muhammad Aksam Iftikhar

**Affiliations:** 1 Department of Electrical and Computer Engineering, Lahore Campus, COMSATS University Islamabad, Islamabad 54000, Pakistan; 2 Department of Computer Science, Lahore Campus, COMSATS University Islamabad, Islamabad 54000, Pakistan

**Keywords:** diabetes mellitus, blood glucose prediction, forecasting, long short-term memory

## Abstract

Diabetes Mellitus, a metabolic disease, causes the body to lose control over blood glucose regulation. With recent advances in self-monitoring systems, a patient can access their personalized glycemic profile and may utilize it for efficient prediction of future blood glucose levels. An efficient diabetes management system demands the accurate estimation of blood glucose levels, which, apart from using an appropriate prediction algorithm, depends on discriminative data representation. In this research work, a transformation of event-based data into discriminative continuous features is proposed. Moreover, a multi-layered long short-term memory (LSTM)-based recurrent neural network is developed for the prediction of blood glucose levels in patients with type 1 diabetes. The proposed method is used to forecast the blood glucose level on a prediction horizon of 30 and 60 min. The results are evaluated for three patients using the Ohio T1DM dataset. The proposed scheme achieves the lowest RMSE score of 14.76 mg/dL and 25.48 mg/dL for prediction horizons of 30 min and 60 min, respectively. The suggested methodology can be utilized in closed-loop systems for precise insulin delivery to type 1 patients for better glycemic control.

## 1. Introduction

The ability of the human body to control imbalanced blood glucose (BG) levels is administered by the pancreas. When the glucose level in the bloodstream increases beyond the normal range, the pancreas senses this and instructs the *β*-cells to release insulin. As a result, excess sugar begins to be stored in fat cells, keeping the blood glucose in the normal range. In contrast, the low sugar level in the bloodstream is handled by *α*-cells, which release glucagon hormone. This hormone dictates the liver releasing glucose into the bloodstream, maintaining BG levels in the safe range. Diabetes mellitus (DM) is a medical condition in which the human body loses this closed-loop control partially or completely. In Type-1 diabetes, the human pancreas loses its ability to produce insulin to regulate elevated sugar levels. 

In Type-2 diabetes, the patient becomes insulin resistant, or the insulin produced becomes ineffective at decreasing the high BG levels. In comparison to other diseases, diabetics have a higher prevalence of illness and mortality. Regardless of its present incidence and burden, i.e., 415 million adults, this disease is projected to affect 642 million by 2040 [1]. In 2015, it was estimated that there were 415 million people with diabetes aged 20–79 years, 5 million deaths attributable to diabetes, and a total global health expense due to diabetes of 673 billion US$. This figure was reported to be 760 billion dollars in a 2019 survey [2]. Since no established cure exists for diabetes, disease management is the only option. Efficient glycemic control requires constant monitoring of BG levels, sufficient physical activity, precise insulin delivery, and appropriate diet intake. In this regard, future estimation of blood glucose levels is the most important aspect for a patient. A realistic assessment of BG levels can help patients avoid fatal glycemic conditions such as hypo-glycemia (BG < 70 mg/dL) or hyper-glycemia (BG > 180 mg/dL). The prediction of blood glucose levels is quite a complex task due to its non-linear nature and multiple confounding factors. Although a number of factors affect BG levels, the few important ones include previous BG values, dietary intakes, insulin infusion, and the physical activity of a patient [3]. 

**Figure 1:**
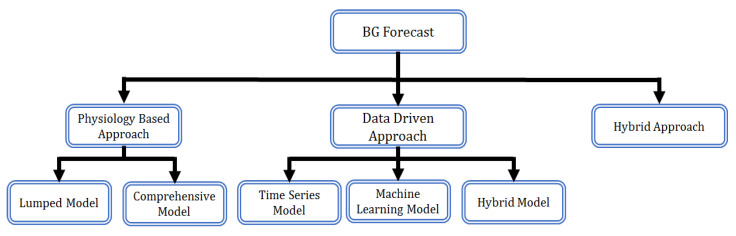
Blood glucose prediction approaches.

BG prediction methodologies can be categorized into three types: Physiology-based (knowledge-based), data-driven (empirical-based), and hybrid. The physiological model requires comprehensive knowledge of individual underlying physiological mechanisms. It divides the person’s BG metabolism into three different regulatory sectors: BG dynamics, meal absorption dynamics, and insulin dynamics [4]. Each one is modeled using a variety of mathematical (differential) equations and probabilistic frameworks. The physiology-based approach is primarily categorized into two: The lumped (semi-empirical) model and the comprehensive model. In contrast to the physiology-based approach, the data-driven strategy relies on the person′s self-recorded historical data and needs little knowledge of the underlying physiological mechanism; thus, referred to as the “black box” approach. Neural networks (NNs) and autoregressive (AR) models are the most common examples of this type of approach. A data-driven approach can generally be divided into three models: A time series model, a machine learning model, and a hybrid model. Hybrid models, as the name suggests, are a combination of both physiological and data-driven models. The blood glucose prediction approaches are illustrated in Figure 1.

Historically, efforts have been made to employ statistical approaches for the prediction of blood glucose levels. A multivariate statistical modeling approach was employed using a latent variable for short-term blood glucose forecasting in T1DM patients [5]. Another study presented a comparative analysis of various machine learning techniques with classical time series forecasting methods such as auto-regression with Exogenous input (ARX) for glucose level prediction [6]. Event detection such as hypo/hyperglycemia has also been an interest of researchers for T1DM patients [7]. In addition to BG level forecasting, the study [8] presented the efficiency of detecting hypo/hyperglycemic events in Type 1 diabetic patients. In another study, the application of the XG-Boost algorithm was proposed for glycemia prediction [9]. The authors in the study [10] developed a model of multi-horizon glucose level prediction with alerts for the hypoglycemia condition.

Recently, machine learning and deep learning applications for blood glucose prediction in patients with Type 1 and Type 2 diabetes have gained a lot of attention. A recurrent neural network (RNN) was employed for blood glucose level forecasting with the provision of forecast certainty [11]. A personalized BG level forecasting model was proposed based on a convolutional neural network (CNN) by converting the regression task into a classification task [12]. Another study [13] proposed a personalized deep learning framework for the prediction of BG distribution over the prediction horizon of 30 and 60 min in T1DM patients. Authors in the study [14] worked on the development of a multitask learning [15] approach using a convolutional recurrent neural network MTCRNN for short-term personalized blood glucose forecasting. Multitask learning allows for learning from the data of all available diabetic subjects who took part in the research. 

The authors of another study [16] proposed the rectification of a sensor defect for CGM readings using the Kalman filter smoothing technique [17]. The prediction of blood glucose levels using a stacked Long-short term memory-based RNN model was presented. The proposed work on sensor fault rectification is prone to flaws since the target labels were modified in lieu of sensor noise rectification.

Deep learning approaches have recently played a significant role in increasing blood glucose forecasting accuracy. However, the need to furnish an optimized deep learning model, for multi-horizon prediction, is not over and requires more research. In addition to the learning model, the discriminative feature representation of sensor data is equally vital to establish an efficient and robust prediction model. Pre-processing of sensor data including normalization, interpolation, and filtering has been performed. However, the discriminative feature transformation of sensor data is rare in the literature. 

In this research work, a deep learning technique based on multi-layer Long Short-Term Memory for blood glucose prediction of T1DM patients is presented. The pre-processing of sensor data is performed, including sampling consistency, interpolation to fill missing samples, and filtering. Moreover, to achieve lower forecasting errors, the event-based features are transformed into time-stamped samples. The Ohio T1DM dataset published by the University of Ohio for the BGLP challenge in 2018 is used. It contains eight weeks of data from six T1DM patients including a variety of physiological features along with event-based information such as meal and insulin injection. The contributions of this research work are as follows:

- Pre-processing of the T1DM dataset is performed including the incorporation of time-consistency in features as per the target values, interpolation for missing values, and filtering to achieve smoothing.
- Based on the relation with blood glucose levels, two event-based features, namely, meal and insulin, are transformed into continuous features, which led to improved accuracy.
- The LSTM and Bi-LSTM-based RNN models are developed and optimized to achieve minimum prediction error for blood glucose levels.
- The proposed models outperformed the state-of-the-art methods for the prediction horizons of 30 and 60 min.

The paper is organized as follows: Section 2 presents the dataset and the pre-processing. Feature transformation is explained in Section 3. The evaluation strategy is discussed in Section 4. The learning algorithm is presented in Section 5. Section 6 presents the results and discussion. Finally, the conclusion is added in Section 7. 

## 2. Dataset and Pre-Processing

### 2.1. Dataset

**Figure 2:**
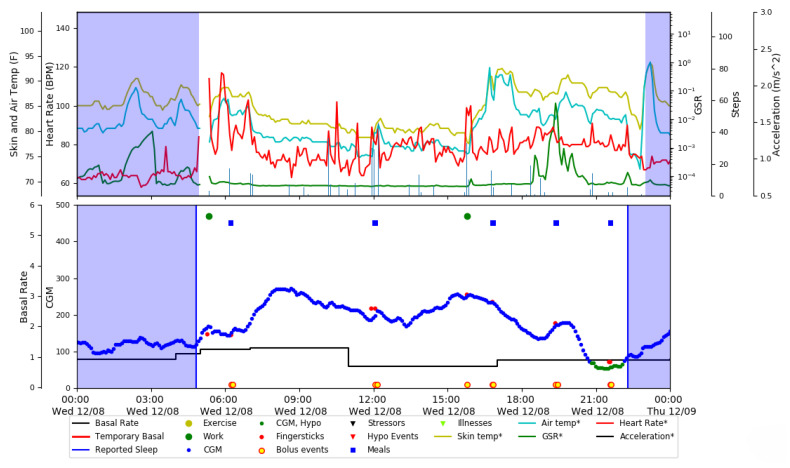
Feature visualization using data of T1DM patient ID 570.

The Ohio T1DM dataset [18] published by the University of Ohio is used in this research. It is a great source of blood glucose forecasting research due to the fact that it provides a diverse range of characteristics associated with Type 1 diabetes patients, which, according to the most recent literature on diabetes [3], affect blood glucose levels. The dataset was released in two phases: In 2018 and in 2020. Each release contains data from six T1DM patients who were on insulin therapy wearing a fitness tracker and Medtronic’s insulin pump with continuous glucose monitoring (CGM). In addition, the self-reported life events from these contributors were also recorded via a smartphone application. The data recorded for each individual includes CGM sampled at 5 min intervals, finger stick blood glucose levels for self-monitoring, insulin doses (both basal and bolus), self-reported time of physical activity, work, stress, sleep, and illness, and physiological information from a fitness tracker such as Galvanic skin resistance (GSR), heart rate, step count, air temperature, and skin temperature. The dataset contains the sensors’ data for a total period of eight weeks. Several data features, such as CGM and heart rate, are recorded at 5 min intervals, whereas others, such as meal and insulin, are recorded only at the time of eating a meal or insulin intake, respectively. An illustrative overview of these features recorded from one of the contributors (ID 570) is illustrated in Figure 2. In each release of the Ohio T1DM dataset, the training data and testing data are already segregated by the provider. Table 1 summarizes the data of six patients in the Ohio T1DM dataset version 2018, including the number of training and testing samples predefined by the data providers. 

### 2.2. Feature Vector

The dataset includes automatic and manual measuring of several parameters forming the feature vector for a patient. The type of feature is periodic and event-based; however, all of them are recorded with a time stamp. The details of recorded features are provided in Table 2. 

### 2.3. Pre-Processing

#### 2.3.1. Time Coherence

The features, as shown in Table 2, are periodic and event-based in nature. Continuous glucose monitoring (CGM)—the vital information—is recorded at a sampling interval of 5 min. However, during sensor replacement, the time coherence breaks. Consequently, upcoming samples are time inconsistent. To avoid this, new samples are added to the data, ensuring a sampling time of 5 min. The net effect is the introduction of many new samples. For example, there exist 10,982 training samples of CGM for patient ID 570. After time coherence, 649 more samples were introduced. Next, since CGM is the most important feature, the rest of the periodic features were also time-aligned with the CGM time stamps. 

#### 2.3.2. Interpolation

**Figure 3:**
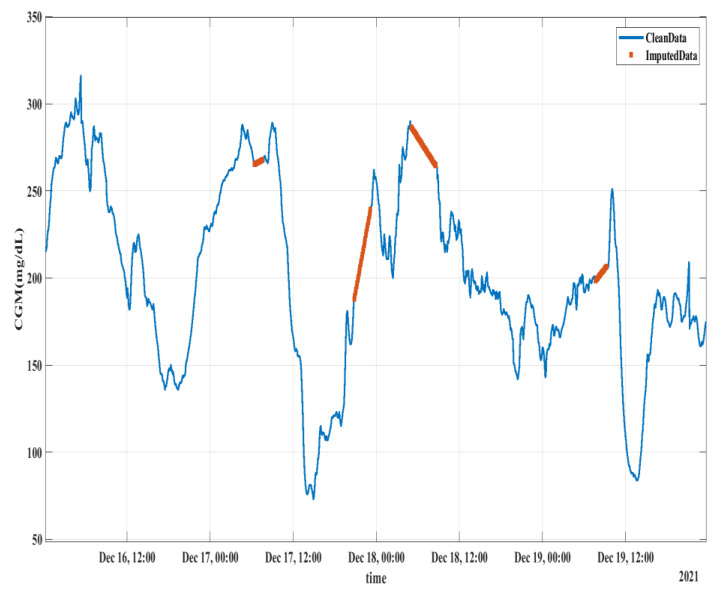
Missing CGM data imputation using linear interpolation. The orange lines represent the interpolated sample data, which were missing in the original dataset.

The criterion for filling new CGM values was based on the forward-filling rule with a limit of 1, i.e., if there is a single new sample, it will be assigned the magnitude of the previous sample. In the case there were 2 or more consecutive new samples, interpolation was performed to fill the samples. Several interpolation techniques including linear, Akima cubic, spline, and shape-preserving methods were used. It was found that linear interpolation learns the best behavior of glucose levels, producing the best prediction results. Figure 3 shows the linear interpolation result of the CGM feature over a period of 4 days. The orange color in Figure 3 shows the interpolated samples, which were empty in the original data. The same interpolation strategy was adopted for the test data. However, for result evaluation, the interpolated samples in the test data were not considered for error calculation.

#### 2.3.3. Median Filtering

**Figure 4:**
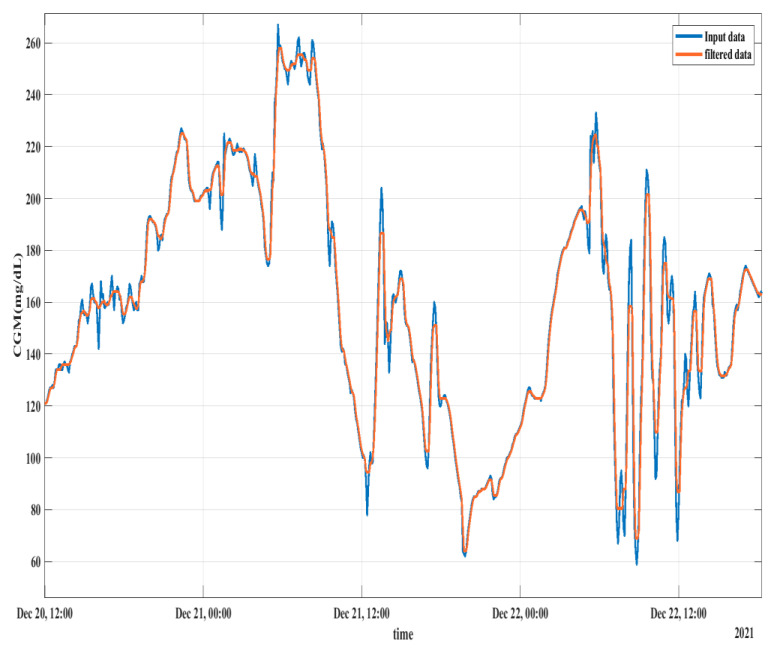
Data smoothing using median filtering.

Observing Figure 3, sharp changes in the CGM profile are visible, which can be regarded as sensor noise. To smooth the profile, a median filter is used replacing the extreme values with the median value as used in an earlier study [19]. Figure 4 shows the CGM profile smoothing using the median filter where the profiles of both the original data and the filtered data are shown. Smoothing the feature profile enables the predictor to learn better. The filtering scheme was only implemented on training data while the test data remained unchanged.

## 3. Feature Transformation

The data include several periodic features such as CGM and some event-based features such as meal and sleep. Since all the periodic features are made time-coherent with the feature, it is beneficial to approximate the event-based features as time-coherent periodic values. The meal and insulin injection are two features recorded at random time stamps. Currently, such features can be treated as binary features where a value of 1 will be observed when a meal was eaten and 0 for the rest of the time. Such values are not only insignificant to the feature vector but also may cause an adverse effect on the learning of the algorithm. Therefore, we propose the transformation of such features into continuous-value observation. This transformation will improve the feature representation for meal and insulin information. To consider those features for blood glucose prediction, they need to be made time-coherent with CGM, which is performed and explained in the following sub-sections.

### 3.1. Carbs from Meal to Operative Carbs Transformation

**Figure 5:**
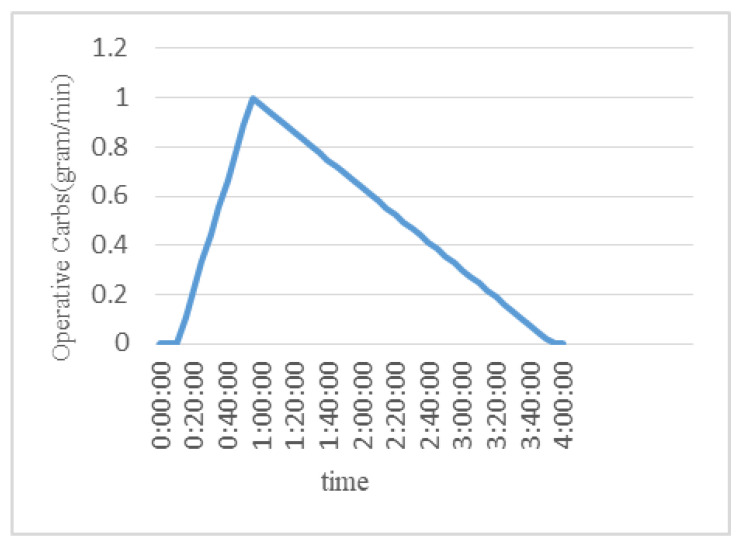
Carbohydrates to the operative carb conversion profile.

One of the data features is the carb intake by patients in the form of a meal, which has a direct impact on blood glucose. The rate at which the carbs affect blood glucose levels depends on their type, as high glycemic index (GI) carbs instantly affect the blood glucose while low-glycemic-index carbs have a slower effect, but the effect remains over a longer period of time. Research [20] revealed that a normal meal starts raising the blood glucose level after 15 min of having the meal. The glucose level approaches its peak in 60 min and then decays at a certain rate such that after 3 h it becomes steady. Based on that, carbohydrate absorption can be converted into operative carbs, which provides a continuous-value feature. The primary objective of this conversion is to determine the effectiveness of carb intake at any certain time in the human body. The conversion model can be estimated by combining the rise and decay profile as shown in Figure 5. 

The operative carbohydrates at any time in the data are estimated using the following procedure:

i Based on the assumption of working at a 5 min reference time scale, the first three samples after taking the meal have zero operative carbs.
ii The operative carbs start rising at a rate of 0.11 (11.1%).
iii At the 12th sample or the 60th minute after having the meal, the value of operative carbs attains its maximum value, which is almost equal to the total amount of carbs.
iv After that, the operative carbs start decreasing at a rate of 0.028 (2.8%). It reaches zero after 3 h.

The mathematical piecewise function of the above-mentioned method can be obtained as follows:

(1) $$C_{\mathrm{op}}\left(t_{s}\right)=\left\{\begin{array}{cc} 0, & \;0\leq t_{\mathrm{meal}}\;\leq 2\\ (t_{s}-t_{\mathrm{meal}+2})/(t_{s}-t_{s-1}\;){*\alpha }_{\mathrm{inc}}{*C}_{\mathrm{meal}}\;, & \;3\leq t_{\mathrm{meal}}<12\\ \left(1-(t_{s}-t_{\mathrm{peak}}\right)/(t_{s}-t_{s-1}\;){*\alpha }_{\mathrm{dec}}){*C}_{\mathrm{meal}}\;, & \;12\leq t_{\mathrm{meal}}<48\; \end{array}\right.$$

where:

$t_{s}$ is the sampling time.
$t_{\mathrm{meal}}$ is the time when the meal is encountered.
$C_{\mathrm{op}}$ is the effective carbohydrates at any given time.
$C_{\mathrm{meal}}$ is the total amount of carbohydrates taken in a meal.
$t_{\mathrm{peak}}$ is the time when $C_{\mathrm{op}}$ reaches its maximum value $\to C_{\mathrm{op}}≅C_{\mathrm{meal}}$.
$\alpha_{\mathrm{inc}}$ is the increasing rate of the curve.
$\alpha_{\mathrm{dec}}$ is the decreasing rate of the curve.

**Figure 6:**
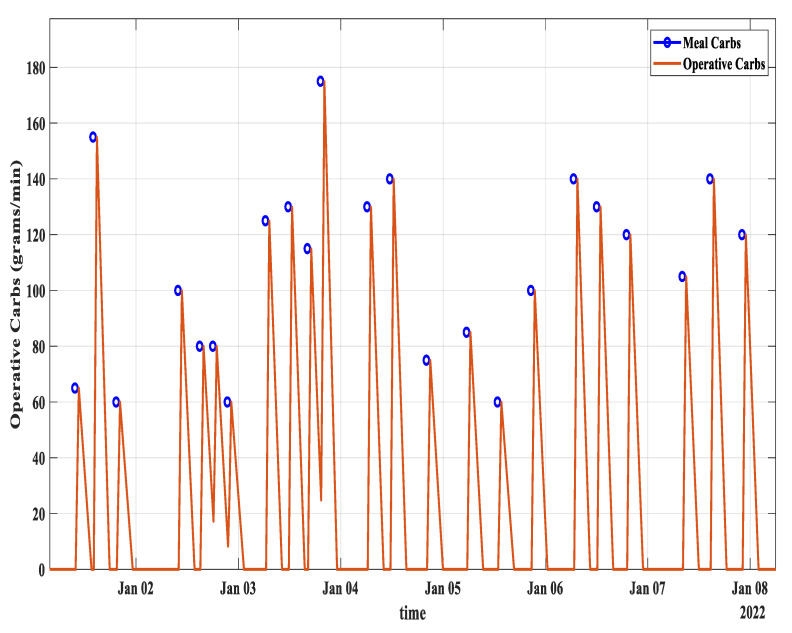
Meal into operative carbs transformation for data of patient ID 570. Blue circles represent the time of meal and the corresponding carbs whereas the brown curves show the transformation into operative carbs.

Figure 6 shows the meal carbs transformation to the operative carbs of patient ID 570.

### 3.2. Bolus Insulin to Active Insulin Transformation

Patients with Type-1 diabetes rely on exogenous insulin delivery [21], which usually has two types, one is rapid-acting insulin named bolus and the second is slow-acting insulin termed basal insulin. Bolus insulin is normally taken before meals to compensate for the effect of a rise in blood glucose. Most insulin-pump users are familiar with the insulin activity curve, as insulin’s effect is time dependent. It takes a short while for insulin to have an effect on the blood glucose level: Normally, it peaks after 1 h and decays gradually over 6 h. The effect of insulin is characterized by the type and brand of insulin used, such as rapid-acting insulin—child, which has a peak time of 65 min, whereas rapid-acting insulin—adult has a peak time of 75 min. The insulin activity remaining or the percentage of active insulin after a bolus insulin injection can be modeled with an exponential decay curve [22] known as insulin on board (IOB). It is the inverse of the insulin activity curve for a given duration of insulin action (DIA).

**Figure 7:**
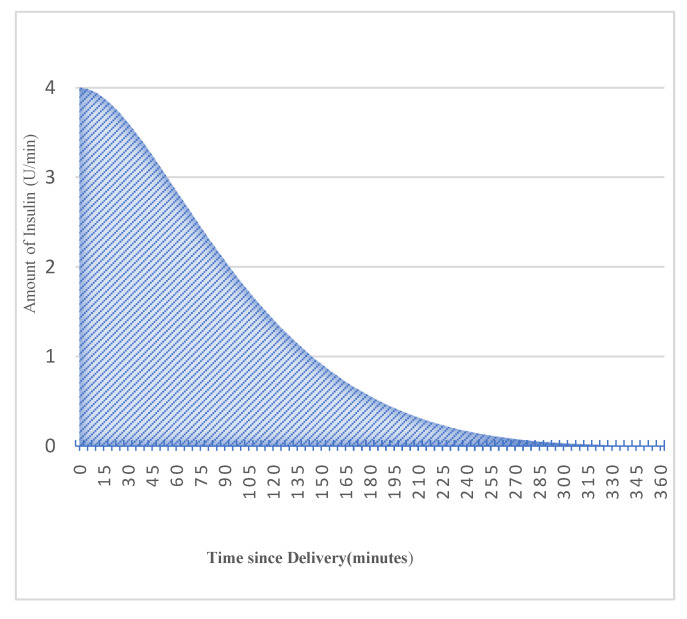
Bolus-to-active-insulin transformation curve.

The transformation of bolus insulin into active insulin is performed. With insulin infusion, the blood glucose is expected to drop based on several aspects such as the insulin sensitivity factor (ISF), amount of dose, and type of insulin. The effect of insulin could be visualized in three different ways, one of which is expected active insulin. We envision the effect of bolus insulin in terms of active insulin. The expected active insulin in the human body at any time is the product of the original insulin delivered and the percent of insulin activity remaining (IOB). Figure 7 shows the transformation of four units of bolus insulin into the respective active insulin. 

The mathematical expression for active insulin can be described as follows:

(2) $$\mathrm{IOB}\left(t_{s}\right)=1-S*\left(1-a\right)*\left\{\left(\frac{t_{s}{}^{2}}{{\tau *t}_{d}*\left(1-a\right)}-\frac{t_{s}}{\tau }-1\right)\;\right.*\left.e^{-\frac{t_{s}}{\tau }}+1\right\}$$

where:

$t_{s}$ is the sampling time.
$t_{d}$ is the total duration of insulin activity.
τ is the time constant of exponential decay.
a is the Rise time factor.
S is the Auxiliary Scale factor.

(3) $$\tau =t_{p}*\left(1-\frac{t_{p}}{t_{d}}\right)/\left(1-2*\frac{t_{p}}{t_{d}}\right)$$

(4) $$a=2*\;\tau /t_{d}$$

(5) $$S=1/\left(1-a+\left(1+a\right)*e^{-\frac{t_{d}}{\tau }}\right)$$

**Figure 8:**
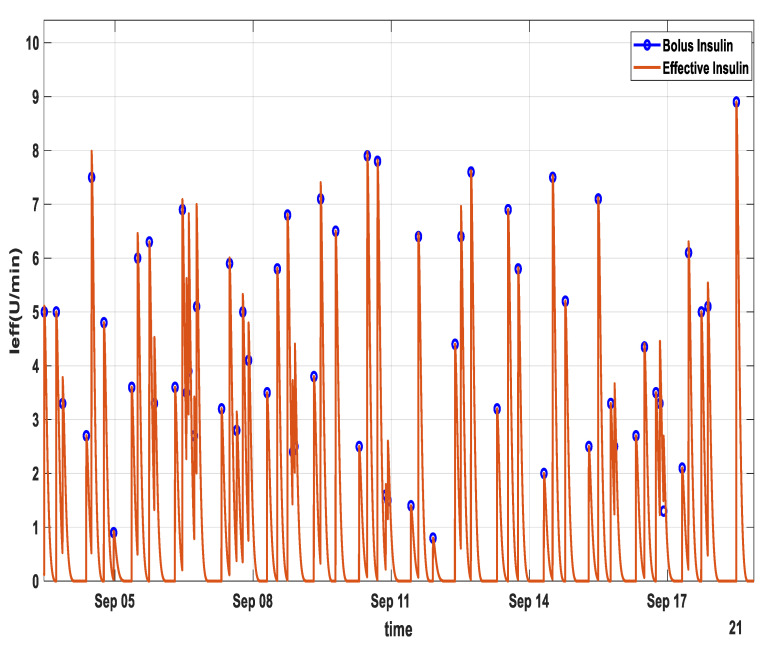
Bolus insulin transformation into active insulin for patient ID 570 over a period of 5 days. Blue circles represent the time and amount of bolus insulin, whereas the brown curves represent the transformation into active insulin over the next 6 h.

Figure 8 shows the bolus insulin intake and the corresponding active insulin for patient ID 570. 

## 4. Evaluation Strategy

### 4.1. Evaluation Metric

The root mean squared error (RMSE) is used to evaluate the performance, as it has been widely used in existing studies. A prediction horizon (PH) of 30 min and 60 min is considered. If the prediction for the ith example made by the model against the actual target $y_{i}$ is denoted as $\bar{y_{i}}$, then RMSE can be expressed as:

(6) $$\mathrm{RMSE}=\sqrt{1/n\sum_{i=1}^{n}{\bar{y_{i}}-y_{i}}^{2}}$$

where *n* is the total number of test samples. It is, once again, worth mentioning that within the test data, only samples that were recorded using the sensor were used to compute the error and not the interpolated samples.

### 4.2. Feature Configurations

**Figure 9:**
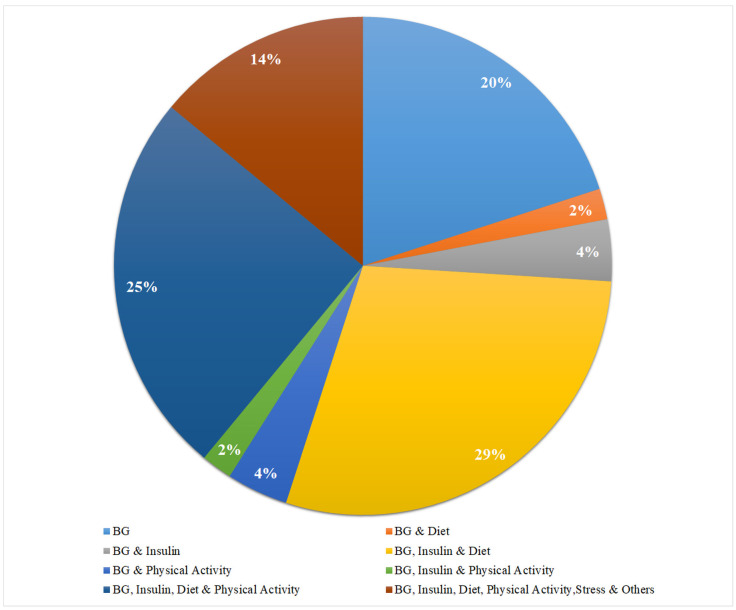
Input Feature set contribution in recent studies for diabetes prediction.

The dataset contains plenty of features; to start with, a recent review study guides the suitable choice based on their historical employment for blood glucose prediction [3]. The adoption of different features in terms of percentage in 624 previous studies is shown in Figure 9. 

Based on widely used features, we opted for BG, diet, and insulin. Overall, three feature configurations are used in this work, as follows:

**Configuration 1 (C-01):** In this scheme, a univariate model is developed using CGM data only.
**Configuration 2 (C-02):** For this scheme, a combination of CGM and operative carbs is used as a feature set.
**Configuration 3 (C-03):** This is the combination of three features: CGM, operative carbs, and active insulin.

**Figure 10:**
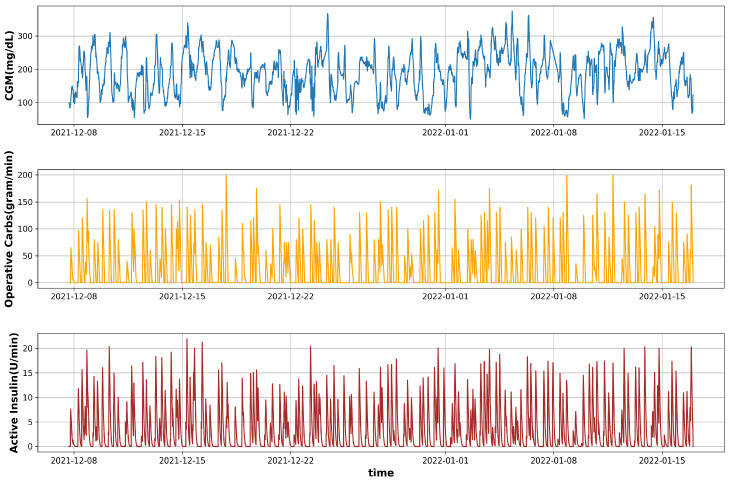
The profiles of CGM, Operative Carbs, and Active insulin in the dataset for patient ID 570.

In addition to the mentioned features, other features such as basal insulin, heart rate, and physiological features were also used; however, the performance was inferior. The complete profiles of the three selected features for this study are shown in Figure 10.

## 5. Learning Model

Continuous blood glucose prediction is a multistep time-series forecasting task, and therefore a sequence-to-sequence learning algorithm is a suitable choice. The class of deep learning models for such a task belongs to recurrent neural networks (RNNs). The long short-term memory (LSTM) model is considered due to its ability to memorize long-term temporal dependencies [23]. In this section, the model architecture is presented first, and then the training and optimization details of the developed model are discussed.

### 5.1. Model Arhitecture

RNNs are a type of neural network that has the capability to learn sequential data. This is because of the provision of feedback paths known as the hidden state *h*. The hidden state can be computed using the following expression:

(7) $$h\left(t\right)=f_{c}\left(Ux\left(t\right)+Wh\left(t-1\right)+b\right)$$

where *h*(*t*) is the current hidden state, $h\left(t-1\right)$ is the previous hidden state,$\;f_{c}$ is a non-linear activation function such as tanh or ReLU (Rectified Linear Unit), *x*(*t*) is the input vector, *b* is the bias term, and *U* and *W* are the weight matrices defined between input-to-hidden and hidden-to-hidden connections, respectively. The standard RNN computes the output through cyclic flow across different hidden layers. It faces the problem of a vanishing gradient due to the short-term memory constraint and thus minimizes the learning. The updated RNN architecture, known as long short-term memory (LSTM), resolves this problem by making use of a memory cell and gates to regulate the flow of information [24]. 

**Figure 11:**
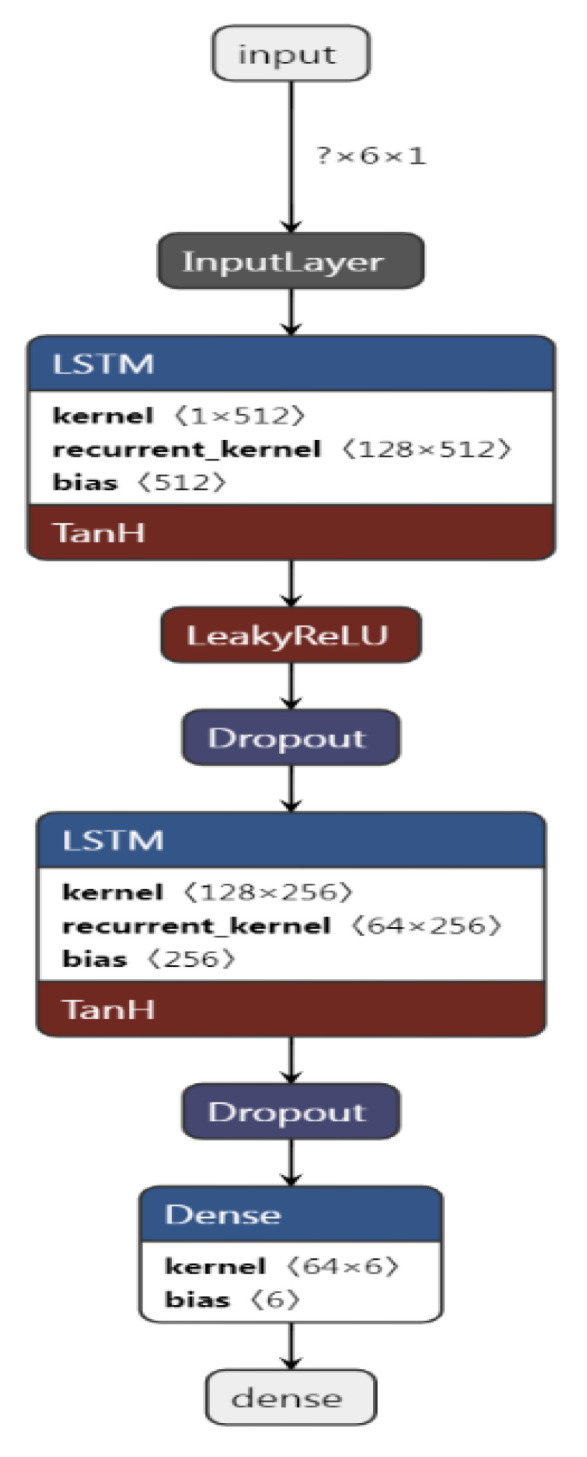
Bi-LSTM model architecture for blood glucose forecasting at 30 min prediction horizon with 6 samples per example and single feature. The size of input data is ‘?× 6 × 1 ′ where ‘?’ represents the number of examples fed to the model.

A two-layer LSTM, i.e., Bi-LSTM, model is developed for this research work, comprising two layers stacked on top of each other. The first hidden layer in LSTM 1 contains 128 neurons followed by a leaky ReLU layer and then a dropout layer to prevent overfitting. The second LSTM layer with 64 neurons is introduced followed by a dropout layer and a dense layer, respectively. The dense layer is the deeply connected output layer of this network, which receives input from all previous hidden layers. The number of units in the dense layer depends on the prediction horizon (PH) for which the model is trained, which are 6 for 30 min PH and 12 for 60 min PH. The developed Bi-LSTM structure for 30 min blood glucose level forecasting is shown in Figure 11. The figure is generated using the software ‘**Netron’**, which is a utility to visualize the trained deep learning models. The model expects data to have the following shape: [*examples, sample per example, number of features*], which the software produced as [? × 6 × 1]. Figure 11 depicts the proposed model architecture that is trained using a single feature with six lag samples (timestamps) to predict the output. 

To understand the input data volume, we consider an example of patient ID 570 with a univariate (one input feature: Glucose level) model, which has the following three-dimensional training and test set shapes:

- Total Number of training samples = 10,982.
- Total Number of training samples after resampling and interpolation = 11,611.
- Number of training samples (80%) = 9288 [1548, 6, 1].
- Number of training examples = 1548 (9288/6).
- Number of test samples (20%): 2322 [387, 6, 1].
- Number of test examples 387 (2322/6).

The training set has 1548 samples with one feature and six lag values. In other words, 9288 timestamps in the training data are converted into 1548 training examples with 6 samples (timestamps) per example. Similarly, there are 387 test examples with 6 samples per test example. The output of the model is 6 samples for a prediction horizon of 30 min as shown in Figure 11.

Similarly, ‘? × 12 × 2′ represents the data input into the model for 60- min prediction (12 samples per example) using 2 features. Likewise, the output of the model for the 60 min prediction horizon will be 12 samples.

In the end, the dense layer produces a vector of 6 values, which represents the prediction for a 30 min horizon. For example:

Input→Output:

[[152. 155. 156. 158. 161. 164.]]=====>[[168. 170. 171. 173. 174. 175.]]

The model takes six previous samples of single features as the input and predicts the six-step-ahead values of the target variable.

Moreover, the vanilla model with a single LSTM layer is also used separately.

### 5.2. Model Training and Optimization

**Figure 12:**
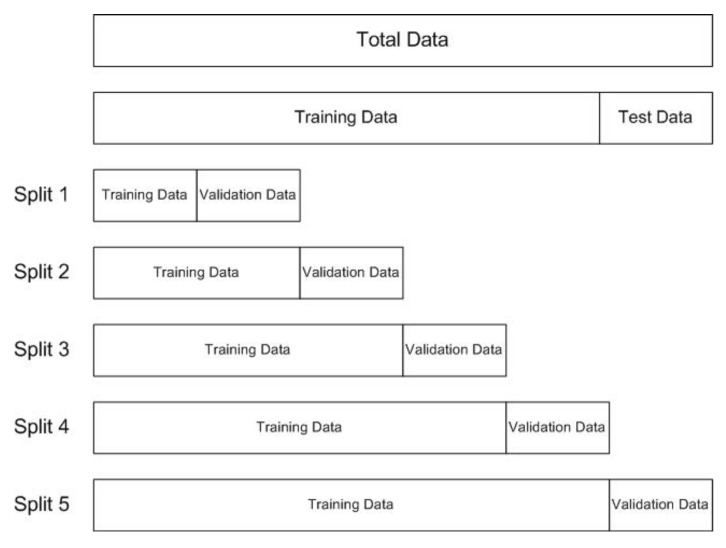
Data splits used for training the model.

**Figure 13:**
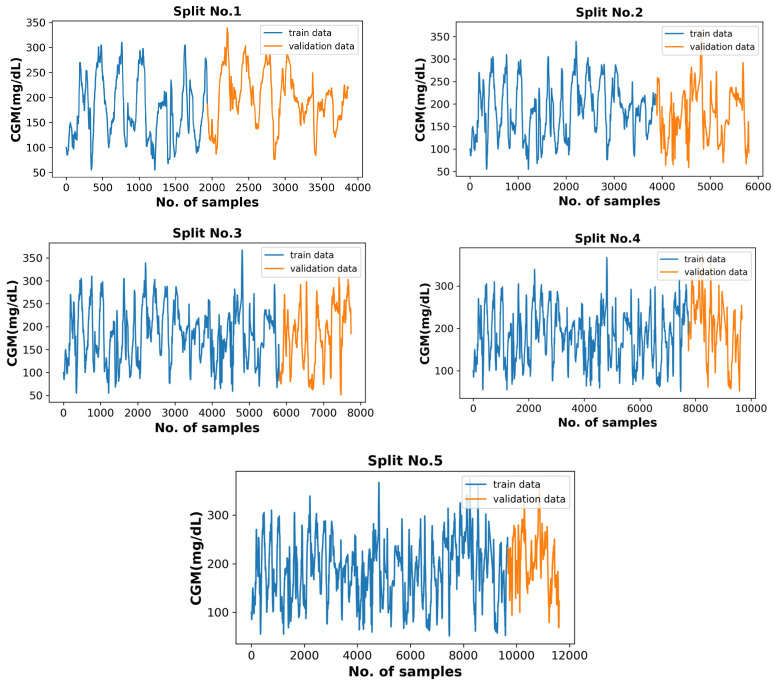
The five combinations of training–validation splits for CGM data of patient ID 570.

The Ohio T1DM dataset includes pre-split files of each contributor as the training data and test data. In this research, the test data are used to finally evaluate the results, while the training data are subdivided for training and validation using the time-series cross-validator [25] provided by the Scikit-learn library of python. It splits the data based on the rolling origin, where each successive training set is a superset of those that come before them. The training data were split into training and validation sets. The five splits were made where the validation data size remains constant, but the training data size increases. For instance, the number of training samples visualized in Figure 12 is 1936, 3871, 5806, 7741, and 9676 in splits 1, 2, 3, 4, and 5, respectively. The number of validation samples remains at 1935 in each split; however, the validation samples themselves change every time. The data-splitting strategy is illustrated in Figure 12. The data for each split are visualized in Figure 13. After normalizing the training and validation data using a standard scalar, the trained model is evaluated using validation data, and the mean score of five splits is recorded. While training, the number of input samples was also altered using the sliding window mechanism with a stride of 1 and a variable window size of half an hour to 6 h. The optimum input sample size was observed as 6, i.e., the last half hour, based on the best mean prediction score. 

As discussed earlier, both vanilla- and Bi-LSTM RNN models are employed; the performance is evaluated based on the blood glucose prediction. The blood glucose regulation system is nonlinear in nature; therefore, to best approximate the input–output relationship, several hyper parameters were adjusted to achieve the optimum results, such as the number of neurons in each LSTM layer, the batch size, the learning rate, and the number of epochs. Different batch sizes including 32, 64, and 128 were used in the experiments where 128 produced the best validation results. Likewise, different combinations of the number of neurons in the LSTM layers were tested and resulted in 128 and 64 neurons in the first and second LSTM layers, respectively. The Adam optimizer was used with an optimized learning rate of 0.001 to train the model. Once all the model parameters are optimized, the final model is evaluated using the walk-forward validation scheme [26] on the test data.

## 6. Results and Discussion

**Figure 14:**
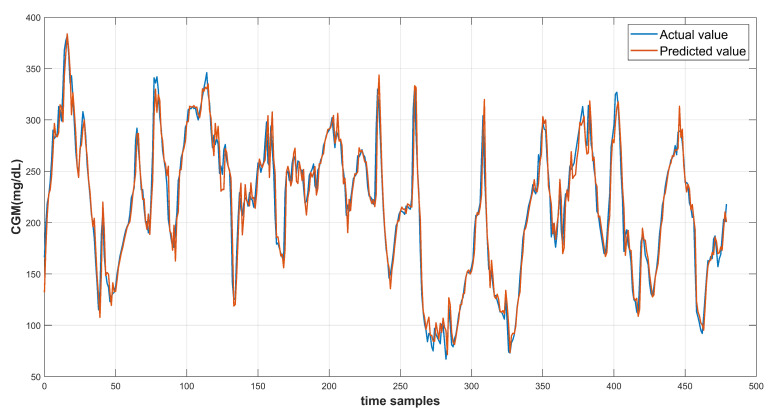
Prediction curve for patient ID 570 at PH of 30 min.

**Figure 15:**
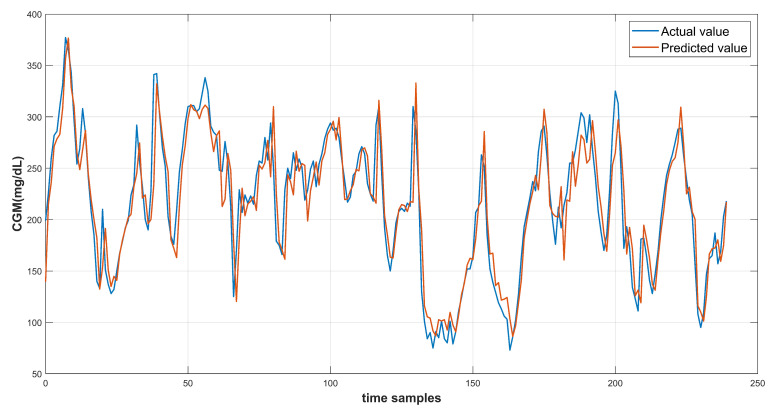
Prediction curve for patient ID 570 at PH of 60 min.

**Figure 16:**
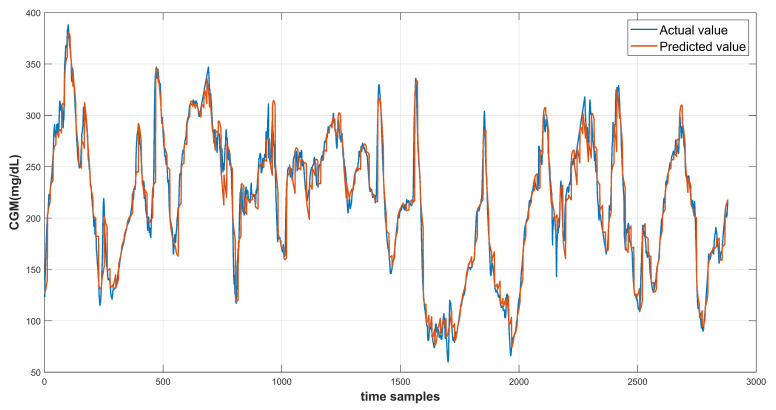
Prediction curve for complete test data of Patient ID 570.

The model results are recorded using the test data provided in the dataset. Each of the three input feature configurations are used to produce the prediction results. The root mean square error is computed to evaluate the model performance. The continuous glucose monitoring (CGM) prediction results of patient ID 570 for 30-min and 60-min prediction horizon are shown in Figure 14 and Figure 15 respectively. Figure 16 shows the complete test data forecasting results for patient ID 570. 

Table 3 shows the model’s prediction error for each of the three feature configurations over 30-min as well as 60-min PH for patient ID 570. The performance of the model with Bi-LSTM shows better results than vanilla LSTM due to its capability of memorizing the long-term temporal dependencies.

The model with an input configuration of C-03 = [CGM, Carbs(Cop), Insulin(Ieff)] produced the best results for the PH of 30 min while configuration C-02 = [CGM, Carbs(Cop)] outperformed for the 60 min PH. Based on these results for patient ID 570, the blood glucose prediction results of other patients were recorded by employing a, similar feature configuration pattern, i.e., configuration C-03 for PH of 30 min and C-02 for 60 min. Table 4 presents the error results produced by the trained model for patients ID 588 and 563. The Bi-LSTM model outperformed the vanilla-LSTM model. A comparison of the proposed model with existing studies for the 30 min prediction horizon is presented in Table 5. It can be observed that the proposed methodology outperformed the state-of-the-art tool except for patient ID 563 where the margin was almost negligible. Moreover, the mean RMSE across the data of three contributors was computed, and the proposed methodology produced the minimum error. 

Among existing studies, for this dataset, several performed blood glucose prediction using the prediction horizon of 60 min. Their results are compared with the proposed model results for the three patients as shown in Table 6. The proposed model produced less prediction error than the state of the art for the prediction horizon of 60 min.

Although the achieved error margin is relatively small with the state of the art, considering the highly non-linear nature of the blood glucose profile, the slight improvement in forecasting becomes significant. The existing studies used features such as insulin and meals as event-based features. However, the proposed methodology incorporates the transformation of event-based features into continuous values, which led to improving the prediction performance. 

## 7. Conclusions

Efficient blood glucose forecasting is critical for continuous monitoring in diabetic patients. This research proposed an efficient method for accurate continuous glucose monitoring. The data of three contributors in the Ohio T1DM (2018) dataset were used for the performance evaluation of the proposed algorithm. Preprocessing of data was performed including interpolation and filtering. Two important event-based input features were transformed into continuous features that are inherent to the critical relationship with the blood glucose level. Recurrent-neural-network-based LSTM models were trained for blood glucose forecasting with prediction horizons of 30 and 60 min. The proposed models outperformed the state-of-the-art tools, achieving an RMSE as low as 14.76 mg/dL and 25.48 mg/dL for prediction horizons of 30 min and 60 min, respectively. This research will help practitioners and patients in closed-loop systems such as artificial pancreas/automated insulin-delivery systems. It will assist people with diabetes in predicting and managing their blood glucose levels, potentially reducing the risk of complications such as hypoglycemia (low blood sugar) or hyperglycemia (high blood sugar). It was observed that our model generated larger errors approximating sharp transitions in the CGM. Moreover, interpolated data may not reflect the true nature of the blood glucose profile. Therefore, efficient approximation of missing samples and the introduction of discriminative features for improved performance are possible future work directions. 

## Figures and Tables

**Table 1:** Ohio T1DM dataset contributor details.

ID	Gender	Pump Model	Sensor Band	Train Samples	Test Samples
559	female	530 G	Basis	10,796	2514
563	male	530 G	Basis	12,124	2570
570	male	530 G	Basis	10,982	2745
575	female	530 G	Basis	11,866	2590
588	female	530 G	Basis	12,640	2791
591	female	530 G	Basis	10,847	2760

**Table 2:** Feature details in Ohio T1DM dataset.

Ser #	Feature Name	Type	Source
1	glucose level	periodic	medtronic Sensor
2	basal insulin	event	self-Reported
3	bolus insulin	event	self-Reported
4	finger stick	event	self-Reported
5	meal	event	self-Reported
6	exercise	event	self-Reported
7	sleep	event	self-Reported
8	work	event	self-Reported
9	hypo Events	event	self-Reported
10	air temperature	periodic	basis Sensor
11	GSR	periodic	basis Sensor
12	heart Rate	periodic	basis Sensor
13	skin temperature	periodic	basis Sensor
14	sleep	periodic	basis Sensor
15	steps	periodic	basis Sensor

**Table 3:** Prediction error using different input feature configurations at 30 min and 60 min PHs for patient 570.

Configuration	RMSE @ PH = 30 min		RMSE @ PH = 60 min	
	Vanilla-LSTM	Bi-LSTM	Vanilla-LSTM	Bi-LSTM
**C-01**	15.43	15.22	26.41	26.10
**C-02**	15.67	15.12	26.12	25.48
**C-03**	15.48	14.76	26.18	25.65

**Table 4:** Prediction error for patient ID 588 and 563.

Patient ID	Configuration	RMSE @ PH = 30 min		RMSE @ PH = 60 min	
		Vanilla-LSTM	Bi-LSTM	Vanilla-LSTM	Bi-LSTM
588	C-02	×	×	30.44	30.17
	C-03	18.29	17.55	×	×
563	C-02	×	×	29.98	29.11
	C-03	18.58	18.14	×	×

**Table 5:** Comparison of prediction RMSE with other studies at prediction horizon of 30 min.

Patient ID	Bi-LSTM (Prposed Work)	[11]	[4]	[5]	[9]
570	14.76	15.94	16.40	18.26	15.73
588	17.55	17.71	19.20	21.69	17.66
563	18.14	18.12	19.00	20.17	18.51
Mean RMSE	16.81	17.25	18.20	20.04	17.30

**Table 6:** Comparison of prediction RMSE with other studies at prediction horizon of 60 min.

Patient ID	Bi-LSTM (Proposed Work)	[18]	[4](Martinsson, et al., 2018)
570	25.48	25.74	28.6
588	30.17	30.45	33.1
563	29.11	30.42	29.9
Mean RMSE	28.25	28.87	30.53

## Data Availability

The data is publically available from the following link: https://pubmed.ncbi.nlm.nih.gov/33584164/.
